# The Minimal Complexity of Adapting Agents Increases with Fitness

**DOI:** 10.1371/journal.pcbi.1003111

**Published:** 2013-07-11

**Authors:** Nikhil J. Joshi, Giulio Tononi, Christof Koch

**Affiliations:** 1 Computation and Neural Systems, California Institute of Technology, Pasadena, California, United States of America; 2 Blue Brain Project, École Polytechnique Fédé ral de Lausanne, Switzerland; 3 Department of Psychiatry, University of Wisconsin, Madison, Wisconsin, United States of America; 4 Allen Institute for Brain Sciences, Seattle, Washington, United States of America; Max Planck Institute for Mathematics in the Sciences, Germany

## Abstract

What is the relationship between the complexity and the fitness of evolved organisms, whether natural or artificial? It has been asserted, primarily based on empirical data, that the complexity of plants and animals increases as their fitness within a particular environment increases via evolution by natural selection. We simulate the evolution of the brains of simple organisms living in a planar maze that they have to traverse as rapidly as possible. Their connectome evolves over 10,000s of generations. We evaluate their circuit complexity, using four information-theoretical measures, including one that emphasizes the extent to which any network is an irreducible entity. We find that their minimal complexity increases with their fitness.

## Author Summary

It has often been asserted that as organisms adapt to natural environments with many independent forces and actors acting over a variety of different time scales, they become more complex. We investigate this question from the point of view of information theory as applied to the nervous systems of simple creatures evolving in a stereotyped environment. We performed a controlled *in silico* evolution experiment to study the relationship between complexity, as measured using different information-theoretic measures, and fitness, by evolving animats with brains of twelve binary variables over 60,000 generations. We compute the complexity of these evolved networks using three measures based on mutual information and one measure based on the extent to which their brain contain states that are both differentiated and integrated. All measures show the same trend - the minimal complexity at any one fitness level increases as the organisms become more adapted to their environment, that is, as they become fitter. Above this minimum, there exists a large degree of degeneracy in evidence.

## Introduction

What is the relationship between complexity and the fitness of evolved organisms, whether natural or artificial? It is often assumed [1]–[4] that while evolving organisms grow in fitness, they develop functionally useful forms, and hence necessarily exhibit increasing complexity [5]. Some, however, argue against this notion [6], [7], pointing to examples of decreases in complexity, while others assert that any apparent growth of complexity with fitness is an admixture of chance and necessity [8], [9]. One reason behind this absence of a consensus is the lack of formal or analytical definitions that permit relating complexity and fitness within a single framework. While many context-dependent definitions of complexity exist [3], [10]–[13], fitness has been less frequently formalized into an information-theoretic framework [14]. One such attempt [15] showed analytically that the fitness gain due to a predictive cue was tightly related to the amount of information about the environment carried by the cue. Another study using an artificial life setup demonstrated that the observed evolutionary trends in complexity, measured as in [16], could be associated with a systematic driving force such as natural selection, but could also result from an occasional random drift away from the equilibrium [17].

**Figure 1:**
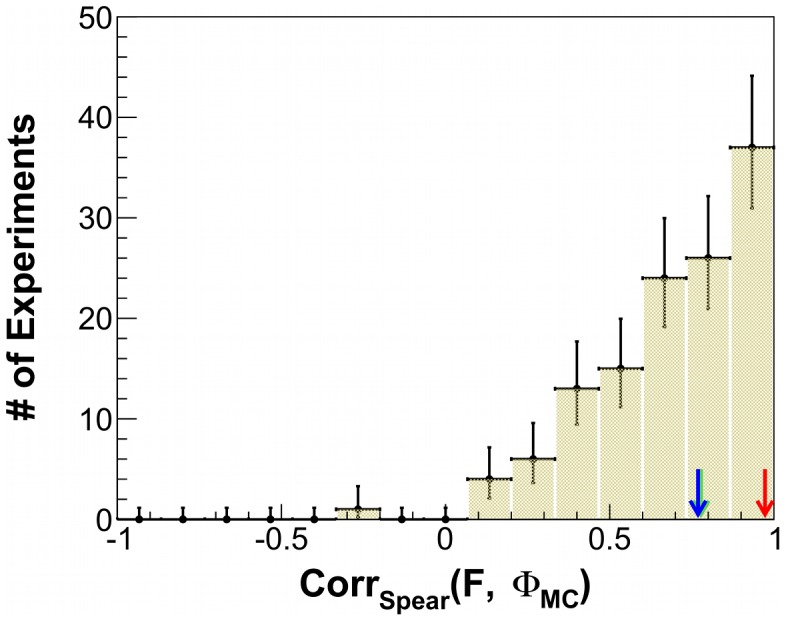
Distribution of the Spearman rank correlation coefficients between 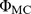 and fitness. The analysis in [18] was repeated several times to obtain Spearman rank correlation coefficients. The distribution for the 126 correlation coefficients shows a very broad spectrum with a mean at 0.69 and a variance of 0.24. The red arrow indicates a value of 0.94 obtained in [18] over 64 evolutionary histories, while the green arrow points to the value of 0.79 obtained for the current 126 histories in the same manner. Error bars are Poisson errors due to binning.

Recently, a computer model of simple animats evolving in an environment with fixed statistics, randomly generated mazes that they had to traverse as quickly as possible (Fig. 1), reported [18] that the complexity of their brains was strongly correlated with their fitness. Using integrated information of the main complex, 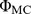 (defined in the latter part of this work), as a measure of complexity, Spearmans rank correlation coefficient between complexity and fitness was 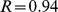. However, no specific relation between these two quantities was established.

In all experiments - and also in our setup - the evolutionary change takes place via two mutually disjoint processes, namely a purely stochastic mutation of the genome followed by a selection process. The stochastic nature of the genetic mutation allows us to equate ensemble-averages over many evolutionary histories to the time-averages over a single history, provided sufficient time has passed for an equilibrium to be established locally. By exploiting this ergodicity, we could greatly scale up the statistic from our evolutionary runs. This enabled us to reproduce the simulations of Edlund *et. al.* [18] for 126 new evolutionary histories (see below) for a more extensive analysis. We obtained a very broad distribution of Spearmans rank correlation coefficients between fitness and 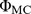, with a mean of 0.69 and a variance of 0.24 (Fig. 1). Even though the distribution shows a tendency for high values, the broad variance hints towards the presence of an uncontrolled, noisy factor that lessens the correlation.

Most information-theoretic definitions of functional or structural complexity of a finite system are bounded from above by the total entropy of the system. The law of requisite variety of Ashby [19] connects the notion of complexity in a control system with the total information flowing between sensory input and the motor output, given by the corresponding sensory-motor mutual information (SMMI) [20]. This relation provides a convenient tool for studying the connection between evolved complexity and fitness. Here, we probe the relationship between fitness and the SMMI in the context of 10,000s of generations of evolving agents, or animats, adapting to a simulated environment inside a computer [18]. In addition to SMMI, we compute three other measures of complexity: the predictive information [12], the state-averaged version of *integrated information* (or 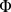 [21]) of a network of interacting parts using the minimal information partition (MIP) as well as the atomic version of 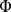, also known as stochastic interaction [22], [23]. We relate all four measures to the extent to which these artificial agents adapt to their environment.

## Results

**Figure 2:**
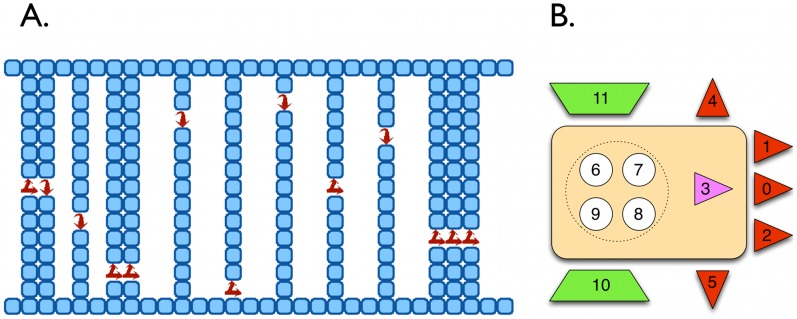
Experimental setup for evolving a population of agents under natural selection in an environment with fixed statistical properties. **A**. A section of the planar maze that the animats have to cross from left to right as quickly as possible. The arrows in each doorway represent a door bit that is set to 1 whenever the next door is on the right-hand-side of the current one and set to 0 otherwise. **B**. The agent, with 12 binary units that make up its brain: b0–b2 (retinal collision sensors), b3 (door-information sensor), b4–b5 (lateral collision sensors), b6–b9 (internal logic), and b10–b11 (movement actuators). In the first generation of each evolutionary history, the connectivity matrix is initiated to be random. The networks for all subsequent generations are selected for their fitness. Taken from [18] with permission from the authors.

In order to test the relationship between the SMMI and the fitness of an agent undergoing adaptation in a static environment, we performed an *in silico* evolution experiment, in which the agent needs to solve a particular task without altering the state of the environment. Our experimental setup is similar to that pioneered by Edlund and others [18], where simple agents evolve a suitable Markov decision process [24], [25] in order to survive in a locally observable environment (described in detail in the Methods section). Agents must navigate and pass through a planar maze (Fig. 2A), along the shortest possible path connecting the entrance on the left with the exit on the right. At every maze door, the agent is instructed about the relative lateral position of the next door with respect to the current position via a single bit (red arrows in Fig. 2A) available only while the agent is standing in the doorway. In effect, an agent must evolve a mechanism to store this information in a one-bit memory and use it at a future time, optimizing the navigation path. For this purpose, the agent is provided with a set of internal binary units, not directly accessible to its environment.

The evolutionary setup, based purely on stochastic mutation and driven by natural selection, allows us to monitor trends in the complexity of the brain of the agents. Our experiment consists of data collected over 126 independent evolutionary trials or histories, where each evolutionary history was run through 60,000 generations. The evolution experiment was carried out using one randomly generated *test* maze, which was renewed after every 100th generation. Frequent renewal of the test maze confirms that each generation of animats does not adapt to a particular maze, by developing an optimal strategy for that particular maze, but enforces evolving a general rule to find the shortest path through the maze. For examples of this evolution, we refer the readers to the movies S1, S2, S3 in the supplementary material.

After every 1000th generation, we estimate the SMMI and complexity in terms of the *predictive* and *stochastic interaction*, and *information integration* of the network evolved so far. To systematically monitor the evolution of network connectivity, we use the data along the line-of-descent (**LOD**) of the fittest agent resulted after 60,000 generations. To reduce the error in fitness as well as complexity estimation, we generated 20 random mazes each time over which performance of an agent is tested to calculate fitness. SMMI and other complexity measures are calculated using the sensory-motor data collected while the agent was navigating through these mazes.

### The Sensory-Motor Mutual Information

The *mutual information* between two variables 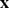 and 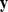 is given by
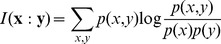
(1)and is a measure of statistical dependence between the two variables [26]. Note, that throughout this work, a boldface symbol such as 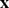 signifies a system (or subsystem) variable, while a particular state of the variable is denoted as a regular-face-type 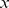, sometimes subscripted as per context as 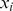. In particular, the SMMI for an agent connectome is evaluated as
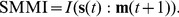
(2)This corresponds to the average information transmitted from the sensors at time 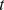, affecting the motor state at one time step later. Our definition of SMMI is a variant of the predictive information used in studies [27], [28] involving a Markovian control system or autonomous robots where sensory input variables 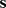 and motor or action variables 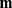 can be distinguished [18]. Depending on whether or not the state-update mechanism uses feedback or memory, these definitions may differ from each other.

**Figure 3:**
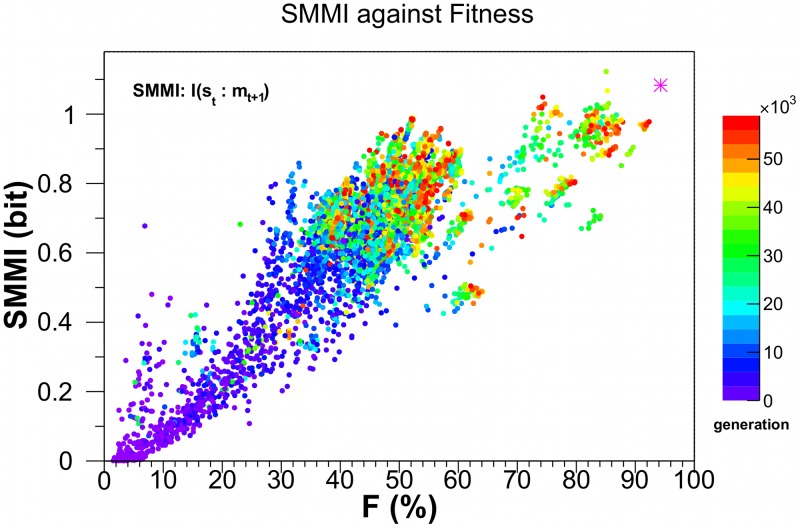
The sensory-motor mutual information, SMMI, as a function of fitness. Along each of 126 evolutionary histories the line-of-descent (**LOD**) of the fittest agent after 60,000th generation is traced back. Absence of cross-over in the evolution confirms that only one agent lies on LOD in every generation. SMMI is calculated every 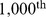 generation for the agent along the LOD. The data is color-mapped according to the number of generation the agent belongs to. The magenta star at 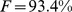 correspond to SMMI of 1.08 bits for *Einstein* - an optimally designed, rather than evolved, network that still retains some stochasticity. Note that SSMI is bounded from above by 2 bits.

Fig. 3 shows the distribution of SMMI calculated for 126 evolutionary histories after every 1000th generation. The data shows increasing lower SMMI values as the fitness of the agents increase.

### Predictive information

The predictive information of a time series, as defined in its original form [12], is a characteristic of the statistic, which quantifies the amount of information about a future state of a system contained in the current state assumed by the system. It can be loosely interpreted as the ability of an external user - as opposed to the intrinsic ability of the system - to *predict* a future state of a system, based on its current state, hence the name *predictive information*. Considering the system as a channel connecting two consecutive states, the predictive information has been proposed as a possible measure of functional complexity of the system. The predictive information of a system 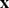 being observed during a time interval of 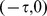 is defined as
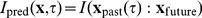
(3a)where 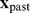 and 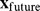 denote the entire past and entire future of the system with respect to an instance at time 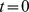.

We here consider the predictive information between one discrete time step, 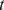 and 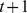, that is for 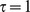 above, or
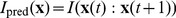
(3b)


**Figure 4:**
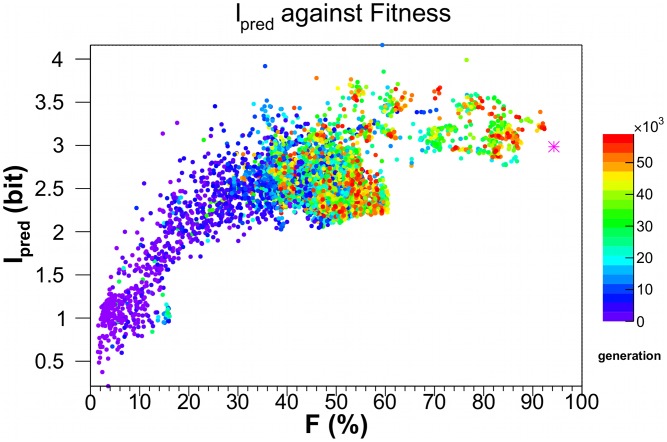
The predictive information, 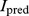, as a function of fitness. 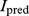
 is calculated for the same networks and in the same manner as in Fig. 3. The magenta star is the 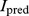 value of 2.98 bits for *Einstein* - an optimally designed agent - with fitness of 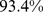. 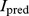 is bounded from above by 12 bits.

Fig. 4 shows the distribution of 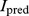 estimated for the evolved agent connectomes along the LODs of the best fit agent at the 60,000th generation in each of the 126 evolutionary histories. Similar to SMMI, 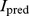 too shows a boundary on the lower side, confirming our expectation of an increasing minimal bound on the complexity with increasing fitness. Indeed, a lower boundary was observed (not shown here) in all cases when we calculated (an approximate) 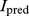 between two states up to 8 time-steps apart.

### Information integration 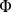


We use the state-averaged version of *integrated information* or 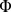 [21] of a network of interacting variables (or nodes) as a measure of complexity and relate it to the degree to which these agents adapt to their environment. The state-averaged version of the integrated information measure 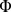 is defined as the minimal irreducible part of the information generated synergistically by mutually exclusive non-overlapping parts or components of a system above the information generated by the parts themselves.

One proceeds by defining a quantity called the effective information
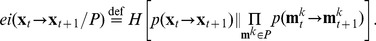
(4)where 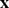 is the whole system and 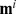 its parts belonging to some arbitrary partition 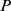. The subscript indices represent temporal ordering of the states. The function 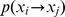 represents the probability of the system making a transition from a state 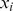 to a state 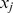. In other words, 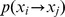 indicates the probability that a variable 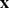 takes a state 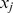 immediately following 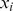. 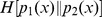 is the Kullback-Leibler divergence or the relative entropy between two probability distributions 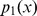 and 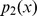, given by
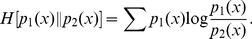
(5)The partition of the system that minimizes the effective information is called *minimal information partition* or MIP. The effective information, defined over the MIP, is thus an intrinsic property of the connectivity of the system and signifies the degree of integration or irreducibility of the information generated within the system. This quantity is called 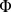 and is given by
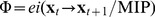
(6)Note that the effective information minimization has a trivial solution, whereby all nodes are included in the same part, yielding a partition of the entire system into a single part. This uninteresting situation is avoided by dividing 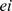 by a normalization factor, given by
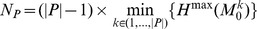
(7)in eq. 4, while searching for a MIP [21]. 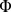, however, is the non-normalized 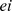 as defined in eq. 6. 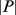 here denotes the number of parts in the partition 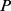, while 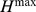 is the maximum entropy.

### The main complex and 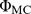


By definition, 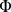 of a network reduces to zero if there are disconnected parts, since this topology allows for a method of partitioning the network into two disjoint parts across which no information flows. That is, the system can be decomposed into two separate sub-systems, rather than being a single system. For each agent, we then find the subset of the original system, called the main complex (MC), which maximizes 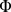 over the power-set of the set of all nodes in the system. This is done by iteratively removing one node at a time and recalculating 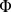 for the resulting sub-network. The corresponding maximal value of the 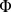 is denoted as 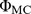.

**Figure 5:**
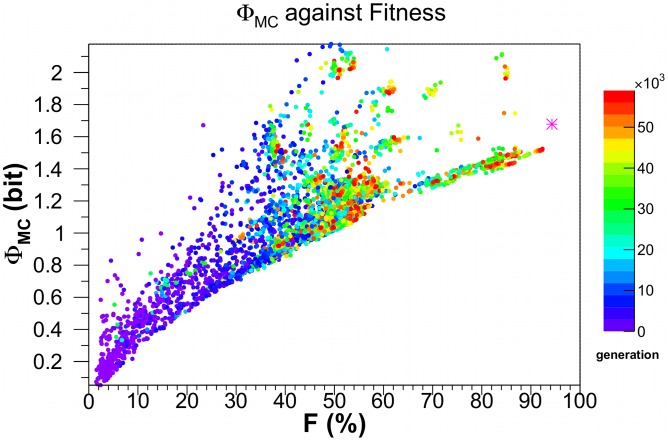
The information integration measure for the main complex, 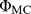, against fitness. 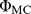
 is calculated for the same networks and in the same manner as in Fig. 3. The magenta star is the 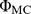 value of 1.68 bits for *Einstein*. 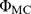 is bounded from above by 12 bits.

Fig. 5 plots 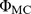 against fitness 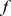. As for the two other complexity measures (SMMI and 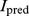), 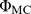 shows a broadly increasing trend with 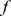. Yet this curve also displays a very sharp lower boundary. That is, the minimal irreducible circuit complexity of our animats, for any one level of fitness, is an increasing but bounded function of the animat's fitness.

### Atomic partition and the 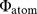


Evaluating 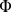 for a system requires searching for MIP of the system - partition that minimizes the effective information for the given dynamical system. MIP search, in turn, necessitates iterating over every possible partition of the system and calculating the 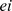 as given in eq. 4. This is computationally very expensive, as the number of possible partitions of a discrete system comprised of 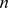 components is given by the *Bell number*, 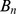, which grows faster than exponentially. As a consequence, determining 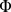 is, in general, only possible for small systems, excluding any realistic biological network [29]. In such cases, a method for approximating either MIP or 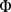 needs to be used.

We denote the effective information calculated over the *atomic* partition 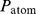 - the finest partition, in which each singleton or elementary unit of the system is treated as its part - by 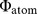. This completely eliminates the need for iterating over the set of partitions of a system. Thus,
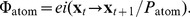
(8a)For a system 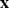 comprised of 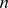 binary units 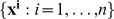 - as is the case with our agents (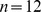) - 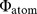 reduces to
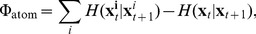
(8b)a measure of complexity, previously introduced as the *stochastic interaction* [22], [23] with the conditional entropy function defined as
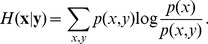
(9)


**Figure 6:**
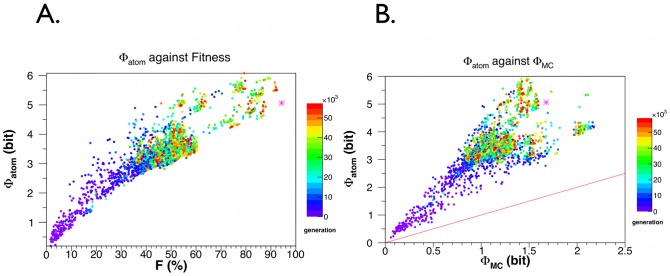
An information integration measure for the atomic partition, 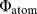, also known as stochastic interaction, as a function of the fitness of the organism. **A**. 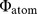 is calculated for the same networks and in the same manner as in Fig. 3. **B**. 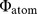 against 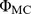 for the same network. The line in red indicates 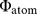 = 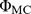. Our data shows that the former is always larger than the latter, as expected from their definitions. The magenta star in both figures are the 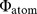 value of 5.06 bits for *Einstein*. 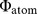 is bounded from above by 12 bits.

The 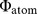 against fitness calculated for the same networks as in Fig. 3 is shown in Fig. 6A. Note, that 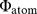, i.e. the integrated information when considering a partition with each node as its own part, is always larger than that of the main complex, 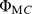, as seen from Fig. 6B. This is expected, since 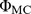 is defined as the minimum over all partitions, which includes the atomic partition over which 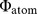 is calculated. In other words, 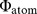 will be necessarily as large as or larger than 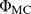.

### Control run

To confirm that selection by fitness is actually necessary to selectively evolve high 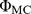 creatures, we carried out two control experiments in which selection by fitness was replaced by random selection followed by stochastic mutation of the parent genome.

In a first control experiment, agents never experienced any selection-pressure, as each new generation was populated by randomly selecting agents from the previous one. Animats unsurprisingly failed to evolve any significant fitness - maximal fitness was 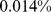 with 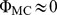.

**Figure 7:**
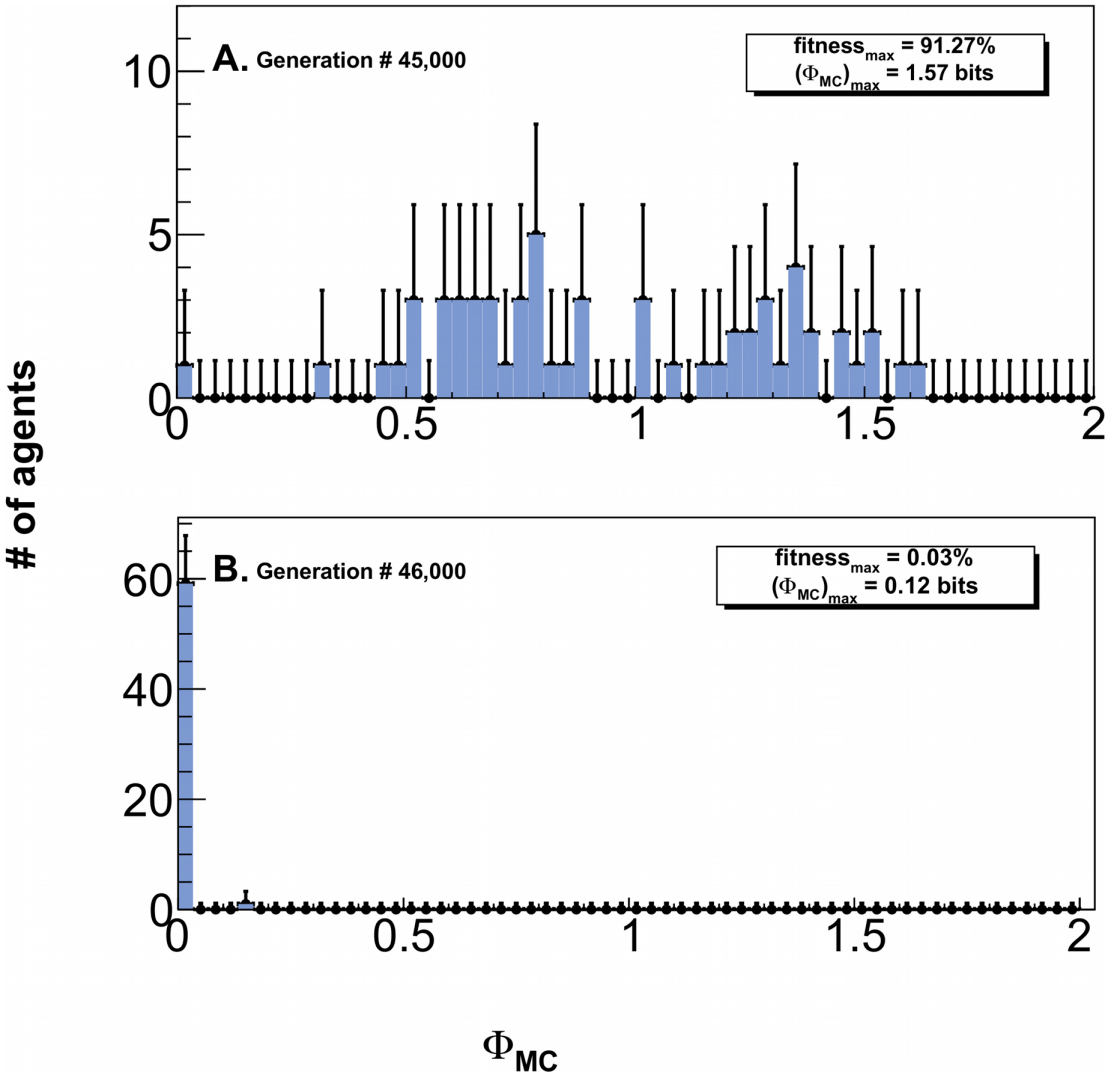
Distribution of evolved complexity with and without selection-pressure for 64 independent histories along their line-of-descent (LOD). **A**. Distribution of 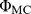 along the LOD using our standard selection based on the fitness after 45,000 generation. Fitness is as high as 91.27%, with a maximal 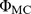 value of 1.57 bit. **B**. Fitness-based selection is then replaced by random selection followed by the usual stochastic mutation of the genome. 1,000 generation later, the population along the LOD has degenerated such that both the fitness as well as 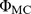 drop to vanishingly small values. The error bars are due to Poisson counting error.

In a second control experiment, organisms evolved as usual for 45,000 generations. This selected for agents able to rapidly traverse through the maze. The resulting 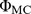 along the LODs over 64 independent runs show a broad distribution, with a maximum of 1.57 bits. The maximal fitness obtained in these runs was 91.27% (Fig. 7A). We then turned off selection via fitness as in the previous experiment. The population quickly degenerated, losing any previously acquired navigational skills within 1,000 generations due to genetic drift - the highest fitness was 0.03%, with an associated 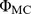 of 0.12 bits (Fig. 7B).

## Discussion

Analyzing various information-theoretical measures that capture the complexity of the processing of the animats as they evolve over 60,000 generations demonstrate that in order to achieve any fixed level of fitness, a minimum level of complexity has to be exceeded. It also demonstrates that this minimal level of complexity increases as the fitness of these organisms increase.

Not only SMMI, but also predictive information 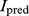 and integrated information 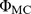 show features similar to SMMI. Indeed our numerical experiments replicate those of [18]. There is a clear trend for integrated information of the *main complex*, 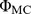 (and also the 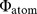 and the predictive information) to grow with fitness 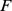, computed relative to a perfectly adapted agent (with 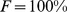). By way of comparison, the fitness of *Einstein*, a near-optimal hand-designed agent within the constraints of our stochastic Markov network, is plotted as a magenta asterisk in Figs. 3–5.

It should be noted, that our terminologies differ slightly from those in [18]; we preserve the original definition of the predictive information [12], termed 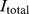 in [18], while our SMMI was originally named predictive information.

Even a cursory inspection of the plots of SMMI, 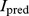 and 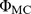 versus fitness reveal a lower boundary - most evident in case of 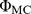 - for any fitness level 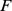. The complete absence of any data points below these boundaries, combined with the high density of points just above them, implies that developing some minimal level of complexity is *necessary* to attain a particular level of fitness. The existence of such a boundary had been previously surmised in empirical studies [1], [2], where complexity was measured crudely in terms of organismal size, number of cell-types, and fractal dimensions in shells.

Conversely, no upper value for complexity is apparent in any of the plots (apart from the entropic bounds of 2 bits for SMMI and 12 bits for 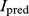 and 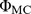 ). That is, once minimal circuit complexity has been achieved, organisms can develop additional complexity without altering their fitness. This is an instance of degeneracy, which is ubiquitous in biology, and which might even drive further increases in complexity [30].

Degeneracy, the ability of elements that are structurally different to perform the same function, is a prominent property of many biological systems ranging from genes to neural networks to evolution itself. Because structurally different elements may produce different outputs in different contexts, degeneracy should be distinguished from redundancy, which occurs when the same function is performed by identical elements. Degeneracy matters not with respect to a particular function, but more generally with respect to fitness. That is, there are many different ways (connectomes) to achieve the same level of fitness, which is exactly what we observe. This provides enough diversity for future selection to occur when the environment changes in unpredictable ways. Curiously, the hand-designed agent, Einstein, has little degeneracy, lying just above the minimal complexity level appropriate for its 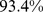 fitness level. In our simulations, any additional processing complexity did not entail any cost to the organisms. This is not realistic as in the real world, any additional processing will come with an associated metabolic or other costs [31]–[33]. We have not considered such additional costs here.

In two control experiments, we showed that selection by fitness is necessary to attain fitness and high circuit complexity. Yet complexity and fitness were neither explicitly connected by construction nor measured in terms of each other. Hence, any network complexity evolved in this manner must be a consequence of the underlying relationship between fitness and complexity. While this complexity is completely determined by the transition table associated with the brain's nodes, its fitness can only be evaluating by monitoring the performance of the agent in a particular environment. This and the fact that all complexity measures studied in this work show similar behaviors support the notion of a general trend between fitness and minimal required complexity.

Thus, complexity can be understood as arising out of chance and necessity [8]. The additional complexity is not directly relevant for survival, though it may become so at a later stage in evolution. On the other hand, a certain amount of redundancy [34], even though not useful for enhancing fitness at any stage, may be necessary for evolutionary stability by providing repair and back-up mechanisms. The previously reported correlation between integrated information and fitness [18] should be understood in this light. High correlation values correspond to data points close to the lower boundary. This strong correlation deteriorates as more and more data lies away from the boundary.

## Methods

### Experimental setup

Our maze is a two-dimensional labyrinth that needs to be traversed from left to right (Fig. 2A) and that is obstructed with numerous orthogonal walls with only one opening or door bored at random. At each point in time, an agent can remain stationary, move forward or move laterally, searching for the open door in each wall in order to pass through. Inside each doorway, a single bit is set that contains information about the relative lateral position of the *next* door (for e.g. arrows in Fig. 2A; a value of 1 implies that the next door is to the right, *i.e.*, downward, from the current door, while a value of 0 means the next door could be anywhere but to the right, *i.e.*, either upward or straight ahead). This *door bit* can only be read by the agent inside the doorway. Thus, the organism must evolve a simple one-bit memory that would enable it to efficiently move through the maze and it must evolve circuitry to store this information in a 1-bit memory.

The maze has circular-periodic boundary conditions. Thus, if the agent passes exit door before its life ends after 300 time steps, it reappears on the left side of the same maze.

Fig. 2B shows the anatomy of the agent's brain with a total of twelve binary units. It comprises a three bit retina, two wall-collision sensors, two actuators, a brain with four internal binary units, and a door-bit sensor. The agent can sense a wall in front with its retina - one bit in front of it and one each on left and right front sides respectively - and a wall on the lateral sides via two collision sensors - one on each side. The two actuator bits decide the direction of motion of the agent: step forward, step laterally right- or left-ward, or stay put. The four binary units, accessible only internally, can be used to develop logic, including memory. The door bit can only be set inside a doorway.

While the wall sensors receive information about the current local environment faced by the agent at each time-step, the information received from the door bit only has relevance for its future behavior. During evolution of the brain of these animats, they have to assimilate the importance of this one bit, store it internally and use it to seek passage through the next wall as quickly as possible.

The connectome of the agent, encoded in a set of stochastic transition tables or hidden Markov modeling units [18], [35], is completely determined by its genome. That is, there is no learning at the individual level.

Each evolutionary history was initiated with a population of 300 randomly generated genomes and subsequently evolved through 60,000 generations. At the end of each generation, the agents ranked according to their fitness populate the next generation of 300 agents. The genome of the fittest agent, or the *elite*, from every generation is copied exactly to the next generation without mutation, while those of other agents selected with probabilities proportional to their fitness are operated over by mutation, deletion and insertion. The probabilities that a site on the genome is affected by these evolutionary operators are respectively 2.5%, 5% and 2.5%.

Evolutionary operators are applied purely stochastically and the selection acts only after the random mutations have taken place. This allows us to relate the fitness-complexity data sampled along each evolutionary line after every 1000th generation - similar to *time averaging* - to that sampled only after 50,000th generation over 64 evolutionary histories - or *ensemble averaged* - as in [36], provided that each evolutionary trial has been run over large enough times confirming exploration of a significant part, if not the entire, of the genomic parameter-space. Fig. 1 shows the distribution of 126 such Spearman rank correlation coefficients calculated per evolutionary trial, with respect to that reported with a red arrow for the 64 evolutionary histories in [18]. The green arrow indicates the rank coefficient value obtained in the same manner for the 126 evolutionary trials from this study.

### Fitness

The fitness of the agent is a decreasing function of how much it deviates from the shortest possible path between the entrance and exit of the maze, calculated using the Dijkstra search algorithm [36]. To assign fitness to each agent as it stumbles and navigates through a maze 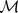 during its lifetime (of 300 time steps), its fitness is calculated as follows: first, the shortest distance to exit, 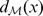 is calculated for every location 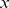 in the maze 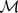 that can be occupied using the Dikjstra algorithm. Each position in the maze receives a fitness score of
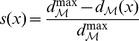
(10)where 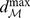 is the maximum of shortest path distances from all positions in 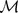. The fitness of an agent over one trial run of 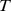 time-steps through 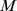 is given by
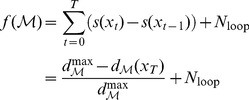
(11)where 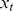 is the position occupied by the agent at time-step 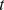 and we use the convention 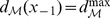 in eq 12, which accounts for the offset due to a non-zero fitness score at the start of the trial, when agent begins navigating 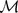 from an arbitrary position, but not necessarily at 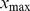 corresponding to 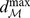. 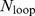 counts how many times the agent has reached the exit in its life and reappeared on the left-extreme of the maze. To reduce the sampling error, final fitness of the agent is then calculated as the geometric mean of its fitness relative to the optimal score from 10 such repetitions.
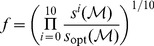
(12)To avoid adaptation bias to any particular maze-design, the maze 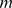 was renewed after every 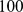 generations.

## Supporting Information

Movie S1
**Typical behavior of an agent from early generations.** The movie shows behavior of an agent from one of the evolutionary trials at 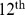 generation in a randomly generated maze. This agent has a fitness of about 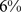. The agent has developed a retina to follow through the doors and always prefers to turn on its right. The top panel is an overview of the agent trajectory throughout the trial, while the lower panel on the left shows a zoomed in area around the agents current position at any time step. The panel on the lower right part displays activity in the Markov units connecting various binary nodes of agent's anatomy. An active node or transition is shown with green color.(FLV)

Movie S2
**An evolved agent traversing through a maze.** The movie shows behavior of an agent from the same evolutionary trial as in **Movie S1**, but after 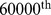 generation. The agent has evolved to a fitness of 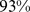 and shows a near-ideal behavior. Due to the stochasticity in the Markov transitions, the agent can make a wrong decision sometimes (for e.g. at around 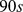 in this movie, it mistakenly turns to left), contributing to its fitness value of less than 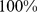. The top panel is an overview of the agent trajectory throughout the trial, while the lower panel on the left shows a zoomed in area around the agents current position at any time step. The panel on the lower right part displays activity in the Markov units connecting various binary nodes of agent's anatomy. A green colored node, state or transition implies current activity.(FLV)

Movie S3
**An optimally designed agent -** ***Einstein*** **, traversing through a maze.** This movie shows the maze-solving capabilities of an agent with optimally engineered connectome. It exhibited a fitness of 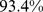 and SMMI, 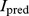 and 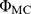 values of 1.08, 2.98 and 1.68 bits, respectively (shown with a magenta asterisk in Figs. 3–5). As in other movies, the top panel is an overview of the agent trajectory throughout the trial, while the lower panel on the left shows a zoomed in area around the agents current position at any time step. The panel on the lower right part displays activity in the Markov units connecting various binary nodes of agent's anatomy. An active node, state or transition are depicted with green color.(FLV)

## References

[1] Bonner J (1988) The evolution of complexity by means of natural selection. Princeton Univ Pr

[2] McShea D (1996) Metazoan complexity and evolution: Is there a trend? Evolution 50: 477–492. 10.1111/j.1558-5646.1996.tb03861.x 28568940

[3] Adami C, Ofria C, Collier T (2000) Evolution of biological complexity. Proceedings of the National Academy of Sciences 97: 4463. 10.1073/pnas.97.9.4463 PMC18257 10781045

[4] Lenski R, Ofria C, Pennock R, Adami C (2003) The evolutionary origin of complex features. Nature 423: 139–144. 12736677 10.1038/nature01568

[5] McShea D (1991) Complexity and evolution: what everybody knows. Biology and Philosophy 6: 303–324.

[6] McCoy J (1977) Complexity in organic evolution. Journal of theoretical biology 68: 457. 599948 10.1016/0022-5193(77)90073-x

[7] Hinegardner R, Engelberg J (1983) Biological complexity. Journal of Theoretical Biology 104: 7–20.

[8] Carroll S (2001) Chance and necessity: the evolution of morphological complexity and diversity. Nature 409: 1102–1109. 11234024 10.1038/35059227

[9] Lenski R (2011) Chance and necessity in the evolution of a bacterial pathogen. Nature Genetics 43: 1174–1176. 22120052 10.1038/ng.1011

[10] Shannon C (1949) Communication in the presence of noise. Proceedings of the IRE 37: 10–21.

[11] Kolmogorov A (1965) Three approaches to the quantitative definition of information. Problems of information transmission 1: 1–7.

[12] Bialek W, Nemenman I, Tishby N (2001) Predictability, complexity, and learning. Neural Computation 13: 2409–2463. 11674845 10.1162/089976601753195969

[13] Tononi G, Sporns O, Edelman G (1994) A measure for brain complexity: relating functional segregation and integration in the nervous system. Proceedings of the National Academy of Sciences 91: 5033. 10.1073/pnas.91.11.5033 PMC43925 8197179

[14] Orr H (2000) Adaptation and the cost of complexity. Evolution 54: 13–20. 10937178 10.1111/j.0014-3820.2000.tb00002.x

[15] Donaldson-Matasci M, Bergstrom C, Lachmann M (2010) The fitness value of information. Oikos 119: 219–230. 25843980 10.1111/j.1600-0706.2009.17781.x PMC4384894

[16] Tononi G, Edelman GM (1998) Consciousness and complexity. Science 282: 1846. 9836628 10.1126/science.282.5395.1846

[17] Yaeger L, Griffith V, Sporns O (2011) Passive and driven trends in the evolution of complexity. arXiv preprint arXiv 11124906.

[18] Edlund J, Chaumont N, Hintze A, Koch C, Tononi G, et al (2011) Integrated information increases with fitness in the evolution of animats. PLoS Computational Biology 7: e1002236. 22028639 10.1371/journal.pcbi.1002236 PMC3197648

[19] Ashby W (1956) An introduction to cybernetics, volume 80. Taylor & Francis.

[20] Touchette H, Lloyd S (2004) Information-theoretic approach to the study of control systems. Physica A: Statistical Mechanics and its Applications 331: 140–172.

[21] Balduzzi D, Tononi G (2008) Integrated information in discrete dynamical systems: motivation and theoretical framework. PLoS computational biology 4: e1000091. 18551165 10.1371/journal.pcbi.1000091 PMC2386970

[22] Ay N (2001) Information geometry on complexity and stochastic interaction. In: MPI MIS PREPRINT 95. Citeseer, pp. 1–33.

[23] Ay N, Wennekers T (2003) Dynamical properties of strongly interacting markov chains. Neural Networks 16: 1483–1497. 14622878 10.1016/S0893-6080(03)00190-4

[24] Puterman M (1994) Markov decision processes: Discrete stochastic dynamic programming. Hobokem, NJ: John Wiley & Sons, Inc.

[25] Monahan G (1982) A survey of partially observable markov decision processes: Theory, models, and algorithms. Management Science 28: 1–16.

[26] Cover T, Thomas J (2006) Elements of information theory, 2^nd^ edition. Wiley Online Library: Wiley-Interscience.

[27] Ay N, Bertschinger N, Der R, Güttler F, Olbrich E (2008) Predictive information and explorative behavior of autonomous robots. The European Physical Journal B-Condensed Matter and Complex Systems 63: 329–339.

[28] Rivoire O, Leibler S (2011) The value of information for populations in varying environments. Journal of Statistical Physics 142: 1124–1166.

[29] Koch C (2012) Modular biological complexity. Science 337: 531–532. 22859475 10.1126/science.1218616

[30] Tononi G, Sporns O, Edelman G (1999) Measures of degeneracy and redundancy in biological networks. Proceedings of the National Academy of Sciences 96: 3257. 10.1073/pnas.96.6.3257 PMC15929 10077671

[31] Zurek W (1989) Thermodynamic cost of computation, algorithmic complexity and the. Nature 341: 14.

[32] Laughlin S, van Steveninck R, Anderson J (1998) The metabolic cost of neural information. Nature neuroscience 1: 36–41. 10195106 10.1038/236

[33] Hasenstaub A, Otte S, Callaway E, Sejnowski T (2010) Metabolic cost as a unifying principle governing neuronal biophysics. Proceedings of the National Academy of Sciences 107: 12329–12334. 10.1073/pnas.0914886107 PMC2901447 20616090

[34] Williams P, Beer R (2010) Nonnegative decomposition of multivariate information. Arxiv preprint arXiv 10042515.

[35] Durbin R (1998) Biological sequence analysis: Probabilistic models of proteins and nucleic acids. Cambridge Univ Pr

[36] Dijkstra E (1959) A note on two problems in connexion with graphs. Numerische mathematik 1: 269–271.

